# Age of red blood cells and outcome in acute kidney injury

**DOI:** 10.1186/cc13045

**Published:** 2013-10-04

**Authors:** Kirsi-Maija Kaukonen, Suvi T Vaara, Ville Pettilä, Rinaldo Bellomo, Jarno Tuimala, David J Cooper, Tom Krusius, Anne Kuitunen, Matti Reinikainen, Juha Koskenkari, Ari Uusaro

**Affiliations:** 1 ANZIC Research Centre, Department of Epidemiology and Preventative Medicine, School of Public Health & Preventive Medicine, Monash University, 99 Commercial Road, Melbourne, VIC 3004, Australia; 2 Intensive Care Unit, Helsinki University Central Hospital, Helsinki, Finland; 3 Department of Clinical Sciences, University of Helsinki, Helsinki, Finland; 4 Finnish Red Cross Blood Service, Helsinki, Finland; 5 Intensive Care Unit, Tampere University Hospital, Tampere, Finland; 6 Intensive Care Unit, North Karelia Central Hospital, Joensuu, Finland; 7 Department of Anesthesiology, Division of Intensive Care, Oulu University Hospital, Oulu, Finland; 8 Intensive Care Unit, Kuopio University Hospital, Kuopio, Finland

## Abstract

**Introduction:**

Transfusion of red blood cells (RBCs) and, in particular, older RBCs has been associated with increased short-term mortality in critically ill patients. We evaluated the association between age of transfused RBCs and acute kidney injury (AKI), hospital, and 90-day mortality in critically ill patients.

**Methods:**

We conducted a prospective, observational, predefined sub-study within the FINNish Acute Kidney Injury (FINNAKI) study. This study included all elective ICU admissions with expected ICU stay of more than 24 hours and all emergency admissions from September to November 2011. To study the age of RBCs, we classified transfused patients into quartiles according to the age of oldest transfused RBC unit in the ICU. AKI was defined according to KDIGO (Kidney Disease: Improving Global Outcomes) criteria.

**Results:**

Out of 1798 patients, 652 received at least one RBC unit. The median [interquartile range] age of the oldest RBC unit transfused was 12 [11-13] days in the freshest quartile and 21 [17-27] days in the quartiles 2 to 4. On logistic regression, RBC age was not associated with the development of KDIGO stage 3 AKI. Patients in the quartile of freshest RBCs had lower crude hospital and 90-day mortality rates compared to those in the quartiles of older blood. After adjustments, older RBC age was associated with significantly increased risk for hospital mortality. Age, Simplified Acute Physiology Score II (SAPS II)-score without age points, maximum Sequental Organ Failure Assessment (SOFA) score and the total number of transfused RBC units were independently associated with 90-day mortality.

**Conclusions:**

The age of transfused RBC units was independently associated with hospital mortality but not with 90-day mortality or KDIGO stage 3 AKI. The number of transfused RBC units was an independent risk factor for 90-day mortality.

## Introduction

Critically ill patients are frequently transfused during their stay in ICU. Around 40% (37 to 44%) of patients receive at least one red blood cell (RBC)-unit transfusion, the mean being 4.6 units per patient during their ICU stay [1,2]. Patients receive transfusions early after ICU admission. For example, 75% of transfused patients receive their first RBC transfusion by day 3 [2]. In general, liberal blood transfusion practice does not appear beneficial in critically ill patients, with the possible exception of those patients with acute myocardial ischemia [3] or severe sepsis and septic shock [4,5].

RBC units undergo changes over time, including several biochemical changes in both RBCs themselves and within the preservative medium [6]. The presence of this storage lesion in RBC units has raised concern for the benefit and safety of RBC transfusions, especially if older RBC units are used. A recent meta-analysis including more than 400,000 patients concluded that in many patient groups including critically ill, cardiac surgery, trauma and pediatric patient populations, the use of older stored blood is potentially associated with a significantly increased risk of death [7]. The majority of the studies in this meta-analysis, however, were retrospective or observational. In addition, three small randomized, controlled trials (RCTs) have shown inconclusive results [8-10]. Finally, a recent large RCT in premature infants found that fresher blood (less than 7 days old) did not reduce mortality or morbidity compared to standard-of-care RBCs [11].

The observational studies included in the meta-analysis of Wang *et al*. reported only short-term mortality (in-hospital, 7-, 28-, and 30-day) except for one study in colorectal cancer patients [7]. The association of the age of RBC transfusions with acute kidney injury (AKI) has not been reported. Thus, the association of age of RBCs with AKI or with long-term mortality in a general ICU population has not been reported so far.

Accordingly, we conducted a prospective observational study as a part of the FINNish Acute Kidney Injury (FINNAKI) study in critically ill patients to evaluate the possible association of the age of RBCs with AKI and with hospital and 90-day mortality. FINNAKI was a prospective study evaluating the incidence, risk factors and 90-day mortality of AKI in unselected critically ill patients.

## Materials and methods

### Data

We conducted a predefined sub-study of the previously published FINNAKI study [12]. FINNAKI was a prospective, observational study comprising 2,901 critically ill patients admitted to 17 Finnish ICUs between 1 September 2011 and 1 February 2012. The patient population of this sub-study included all FINNAKI patients between 1 September 2011 and 30 November 2011.

The Ethics Committee of the Department of Surgery in Helsinki University Hospital gave approval for this study and the use of deferred consent. Signed, informed consent was obtained from the patient or proxy as soon as possible after ICU admission. If informed consent could not be obtained, the Finnish National Institute of Health and Welfare approved the data collection for study purposes from deceased patients.

### Study patients

The study inclusion criteria were: 1) all emergency ICU admissions, and 2) all elective patients with an expected ICU stay of >24 hours. The exclusion criteria were: 1) age less than 18 years, 2) readmission after receiving renal replacement therapy (RRT) during a previous admission, 3) elective ICU admission for less than 24 hours if discharged alive, 4) chronic dialysis, 5) organ donation, 6) no permanent residency in Finland or insufficient language skills, 7) transferred patients included in the study in previous ICU, and 8) intermediate care patients.

### Red blood cell products

In Finland, all RBC units are provided by the Finnish Red Cross Blood Service. RBC products are prepared from anticoagulated whole blood by the buffy coat method. After addition of saline-adenine-glucose-mannitol (SAGM) solution RBCs are leukodepleted by filtration. RBC products comply with European Union directives and Council of Europe standard [13]. In RBC product quality control (n = 2,904, year 2011), the mean amount of hemoglobin was 49.8 g/unit (CI 95% 40.6, 59.0 g/unit) and the number of residual leukocytes 0.03 × 10^6^/unit (CI 95% 0.00, 0.13 × 10^6^/unit). The shelf life of RBC products is 35 days.

### Data collection

The Finnish Intensive Care Consortium prospective database (Tieto Ltd, Helsinki, Finland) served as the source for routine ICU data. The study-specific expansion of the database included study case report forms and calculation of KDIGO (Kidney Disease: Improving Global Outcomes) stage for each patient, continuously based on every measured hourly urine output and plasma creatinine. The daily case report forms were collected from ICU admission to day 5 in the ICU. The data collection consisted of AKI and AKI risk factors, including severe sepsis, using the definition of the American College of Chest Physicians/Society of Critical Care Medicine (ACCP/SCCM) [14]. Each patient was included into the study only once. For readmitted patients, only the admission with the oldest RBCs transfused was included in the analysis. Nine randomly chosen study sites were monitored for the reliability of the data collection with a structured monitoring plan. The dates and donation numbers of all RBC units transfused to study patients during their ICU stay were obtained directly from the participating ICUs. The Finnish Red Cross Blood Services provided information on RBC product details including donation and expiry dates. We obtained data on 90-day mortality from the Finnish Population Register Center.

### Definitions

AKI was defined according to the KDIGO criteria [15]. Renal non-recovery was defined as dependence on RRT at day 90 [16]. The quartiles of transfused patients were defined by calculating the age of all RBCs transfused to the patient. The oldest RBC unit was chosen as the index unit to allocate the patient to the RBC age quartiles [17]. The lowest quartile (Q1) denotes patients with the freshest oldest RBC unit. Q2, Q3, and Q4 denote patients in the second, third and fourth quartiles, respectively. Massive blood transfusion was defined as transfusion of >10 RBC units in 24 hours.

### Statistical analyses

Data are presented as the median and IQR or as absolute number and percentage. We calculated 95% CI for the outcomes. We compared groups using the Mann–Whitney *U*-test for continuous data, and the Fisher exact test for categorical data. We studied factors associated with KDIGO stage 3 AKI during the first 5 days of ICU admission, hospital mortality, and 90-day mortality as dependent variables in separate logistic regression models. Variables were selected for the reported models using the enter method. Covariates included in the model predicting KDIGO stage 3 AKI were: pre-ICU hypovolemia, pre-ICU use of colloids, presence of chronic kidney disease, simplified acute physiology score (SAPS) II (without age and renal components, that is, urine output, serum urea, potassium, HCO3), age, gender, RBC age quartile prior to the highest KDIGO AKI stage and number of RBC units transfused prior to AKI. Patients with KDIGO stage 3 AKI prior to RBC transfusion were excluded from this model. For patients without AKI, we included RBC units transfused within 26 hours from ICU admission, which corresponded to the median time of development of the KDIGO stage 3 AKI. For the models predicting hospital mortality, and separately for 90-day mortality, we generated a propensity score to balance between the patients’ probability of receiving older blood (>14 days in Q2 to Q4) and included patients’ blood groups, study sites, and the numbers of transfused units in the scoring. To study associations with hospital and 90-day mortality, we used the enter method to add covariates in logistic regression analysis. Covariates in these models were: propensity to receive older blood, age, gender, number of transfused RBC units, age quartiles of transfused RBCs (Q2 to Q4 versus Q1) presence of AKI, severe sepsis, acute physiology and chronic health evaluation (APACHE) II diagnosis group, operative admission, emergency admission, disseminated intravascular coagulopathy (DIC), SAPS II without age points, maximum sequential organ failure assessment (SOFA) score during ICU stay and highest lactate value. We also performed Cox regression analysis of 90-day mortality with the same explanatory variables as we had covariates in logistic regression analysis for the hospital and 90-day mortality. In all analyses a *P*-value less than 0.05 was considered statistically significant. Reported *P*-values were not corrected for possible multiple testing. Data were analyzed with SPSS version 19 (SPSS, Chicago, IL, USA).

## Results

Of the 1,811 eligible patients, 665 received at least one RBC transfusion during the ICU stay. In 13 patients, however, the age of RBC units could not be confirmed and they were excluded from the analysis leaving 652 patients (36.6%) in the transfused patient population. During ICU treatment, the transfused patients received 3 (2 to 6) units of RBCs. The patient characteristics according to transfusion status are presented in Additional file 1: Table S1. The median (IQR) age of all RBC units transfused was 14 (11–19) days and the median age of the oldest RBC unit transfused was 18 (14 to 25) days. Of all 3,325 transfusions, 2,334 (70.2%) were given during the first 72 hours of ICU treatment, and the median time from ICU admission to the administration of the oldest RBC unit was 22 (0.2 to 70.0) hours. The times for ICU admission, AKI and RBC transfusions are presented in Additional file 1: Table S2. The patient characteristics according to the quartiles of the oldest RBCs are presented in Table 1.

**Table 1:** Patient characteristics in quartiles according to oldest red blood cell (RBC) transfusion during the ICU stay

	**Q1**	**Q2**	**Q3**	**Q4**	***P*-value**
	**(n = 143)**	**(n = 156)**	**(n = 179)**	**(n = 174)**	
Age, years	66 (58, 76)	66 (57, 75)	65 (54, 74)	66 (55, 75)	0.686
Male gender	82 (57.3%)	102 (65.4%)	114 (63.7%)	113 (64.9%)	
Operative admission	80 (55.9%)	85 (54.5%)	92 (51.4%)	74 (42.5%)	0.067
Emergency admission, n/total	105/143 (73.4%)	114/153 (74.5%)	146/174 (83.9%)	149/172 (85.6%)	0.004
Emergency surgery <1 week	38 (26.6%)	42 (26.9%)	58 (32.45)	49 (28.2%)	0.369
Cardiac or vascular surgery	44 (30.8%)	56 (35.9%)	41 (22.9%)	29 (16.7%)	<0.001
Trauma	9 (6.3%)	6 (3.8%)	16 (8.9%)	13 (7.5%)	0.303
Severe sepsis	34 (23.8%)	50 (32.1%)	69 (38.5%)	69 (39.7%)	0.011
DIC	5/140 (3.5%)	12/151 (7.9%)	13/170 (7.6%)	16/168 (9.5%)	0.239
Acute kidney injury	52 (36.4%)	78 (50.0%)	69 (38.5%)	69 (39.7%)	0.029
Renal replacement therapy	8 (5.6%)	28 (17.9%)	34 (19.0%)	26 (14.9%)	0.004
SAPS II score	35 (27, 44)	36 (30, 50)	40 (28, 55)	38 (31, 53)	0.050
Maximum SOFA score	8 (6, 10)	9 (6, 11)	9 (6, 11)	9 (6, 11)^a^	0.031
Mechanical ventilaiton	110 (76.9%)	132 (84.6%)	149 (83.2%)	132 (75.9%)	0.112
Vasoactive use	111 (77.6%)	124 (79.5%)	142 (79.3%)	130 (74.7%)^a^	0.751
Lactate, mmol/L^b^	1.96 (1.20, 3.40)	2.80 (1.50, 3.40)	2.40 (1.44, 4.60)	2.54 (1.50, 4.77)	0.009
Age of all RBCs, days	11 (9, 12)	14 (11, 15)	17 (13, 20)	17 (12, 26)	<0.001
Maximum age of RBCs, days	12 (10, 13)	15 (14, 16)	21 (19, 22)	29 (27, 32)	<0.001
Transfused units, n	2 (2, 4)	3 (2, 5)	3 (2, 6)	4 (2, 8)	0.001
RBC units over 14 days old, n	0	2 (1, 3)	2 (1, 4)	3 (2, 6)	<0.001
Massive transfusion pre-ICU^c^	8 (5.6%)	9 (5.8%)	7 (3.9%)	15 (8.6%)	0.312
Admission hemoglobin, g/L ^d^	100 (90, 112)	97 (88, 107)	97(88, 110)]	98 (85, 113)	0.544

Results are presented as median (IQR) or number (%). ^a^Data missing for 1 patient in Q4; ^b^highest lactate 24 h preceding ICU admission or on first ICU treatment day, data missing for 11 patients in Q1 and Q2, in 12 patients in Q3, and 17 patients in Q4 (and 40 patients in Q2 to Q4); ^c^transfusion >10 RBC units in 24 hours; ^d^data missing for 11 patients in Q1, 19 in Q2, 27 in Q3, 20 in Q4 (and 86 patients in Q2-4); n, number; RBC, red blood cell; DIC, disseminated intravascular coagulopathy; SAPS II, simplified acute physiology score; SOFA, sequential organ failure assessment.

The incidence of different stages of AKI and administration of RRT, according to quartiles of the oldest RBC unit, are presented in Table 2. In the logistic regression model, only the SAPS II score (without age and renal components) and number of RBCs transfused prior to AKI were independent risk factors for KDIGO stage 3 AKI (Table 3). The number of patients with renal non-recovery was inadequate for analysis as a dependent variable by logistic regression (Table 4).

**Table 2:** Incidence of AKI according to KDIGO staging in transfused patients according to quartiles of oldest red blood cells transfused

	**Quartile 1 (n = 143)**	**Quartiles 2 to 4 (n = 509)**	***P*-value**
**No AKI**	63.6% (55.8, 71.5)	49.5% (45.2, 53.9)	0.003
**Stage 1**	19.6% (13.1, 26.1)	19.6% (16.2, 23.1)	>0.999
**Stage 2**	5.6% (1.8, 9.4)	9.4% (6.9, 12.0)	0.177
**Stage 3**	11.2% (6.0, 16.4)	21.4% (17.9, 25.0)	0.006
**RRT**	5.6% (1.8, 9.4)	17.3% (14.0, 20.6)	<0.001

Results are presented as percentage of patients (95% CI). AKI, acute kidney injury; KDIGO, Kidney Disease: Improving Global Outcomes; RRT, renal replacement therapy.

**Table 3:** Odds ratios with 95% CI from logistic regression analysis of KDIGO stage 3 acute kidney injury

**Variable**	**Odds ratio**	**95% CI lower**	**95% CI upper**	***P*-value**
Age, years	1.005	0.984	1.026	0.628
Female gender	0.586	0.306	1.124	0.108
Chronic kidney disease	1.924	0.737	5.021	0.181
SAPS II score without points for age and renal componenents, points	1.060	1.037	1.084	<0.001
Pre-ICU colloids	1.134	0.615	2.093	0.686
Pre-ICU hypovolemia	1.177	0.625	2.219	0.613
RBC, Q2 to Q4 versus Q1	1.066	0.507	2.238	0.867
Units transfused pre-AKI, number	1.076	1.019	1.135	0.008

In total, 404 patients were included in the model. Hosmer-Lemeshow Chi-square 9.390 *P* = 0.310. Q1 denotes the first and freshest oldest RBC unit quartile, Q2 to Q4 denotes the second to the fourth quartile. SAPS II, simplified acute physiology score, RBC, red blood cell; AKI, acute kidney injury.

**Table 4:** Patient outcomes (quartiles according to the oldest red blood cells transfused)

	**Quartile 1**	**Quartiles 2 to 4**	***P*-value**
	**(n = 143)**	**(n = 509)**	
**ICU length of stay, days**	2.8 (1.6, 4.8)	4.2 (1.9, 8.7)	<0.001
**Renal non-recovery†**	1.4% (0.4, 5.0)	1.2% (0.5, 2.6)	0.689
**Hospital mortality**	10.5% (5.5, 15.5)	20.8% (17.3, 24.4)	0.005
**90-day mortality**	20.3% (13.7, 26.9)	29.9% (25.9, 33.8)	0.026

The results are presented as median and IQR or as percentage with 95% confidence interval. Renal non-recovery is defined as dialysis dependence at 90 days.

**Figure 1:**
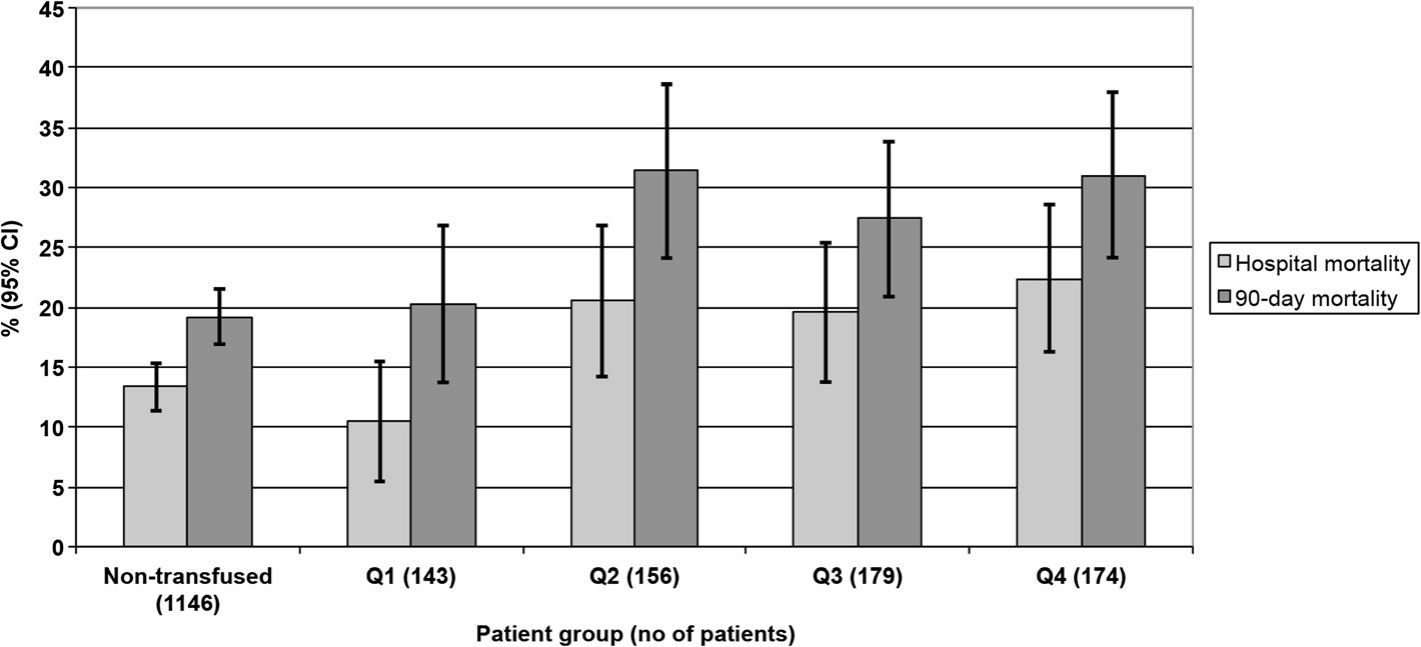
Crude hospital and 90-day mortality in non-transfused and transfused patients according to quartiles (Q) of the oldest red blood cell (RBC) unit.

**Figure 2:**
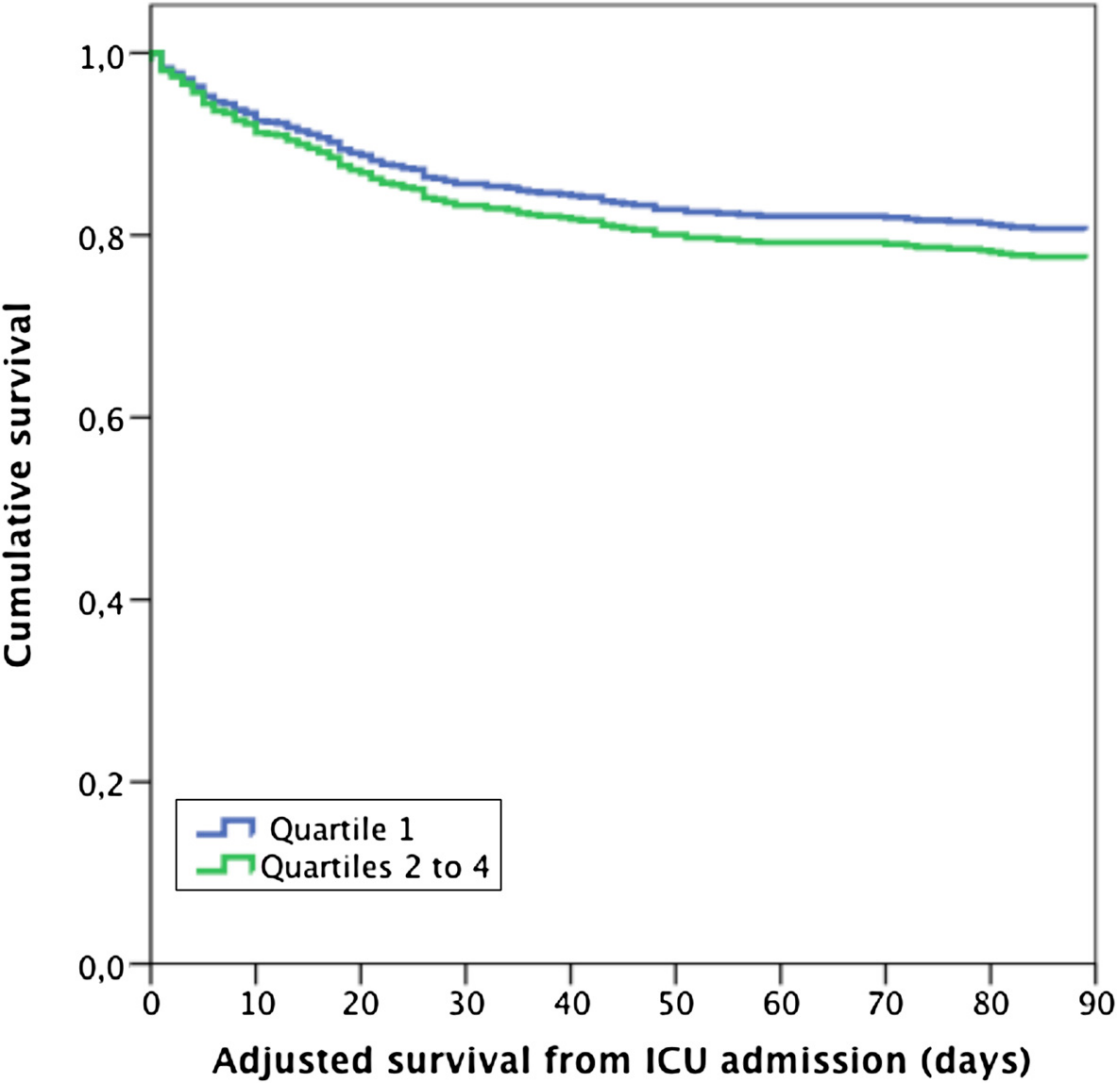
Kaplan-Meier curve (adjusted for baseline variables) for non-transfused and transfused patients according to quartiles (Q) of the oldest red blood cell (RBC) unit.

The ICU length of stay, renal non-recovery, hospital and 90-day mortality rates are presented in Table 4. On logistic regression, patients in RBC age quartiles 2 to 4 had increased risk of hospital mortality compared to patients in the freshest quartile. Age, SAPS II without age points and maximum SOFA score were also associated with increased risk of hospital mortality (Additional file 1: Table S3). In a separate model for 90-day mortality, age, number of transfused units, SAPS II without age points and maximum SOFA score were significant predictors, whereas the age of RBCs was not (Table 5). The hazard ratios from Cox regression analysis are presented in Additional file 1: Table S4. The hospital and 90-day mortality rates in transfused patients according to quartiles of the oldest transfused RBCs are presented in Figures 1 and 2.

**Table 5:** Odds ratios and 95% CI from logistic regression analysis of 90-day mortality

**Variable**	**Odds ratio**	**95% CI**	**95% CI**	***P*-value**
		**lower**	**upper**	
Age	1.043	1.025	1.061	<0.001
Propensity score for receiving >14 day-old RBCs	0.083	0.004	1.791	0.112
Transfused units, number	1.057	1.010	1.106	0.017
Acute kidney injury	0.774	0.472	1.271	0.312
Severe sepsis	0.785	0.478	1.288	0.338
RBC age Q2 to Q4 versus Q1	1.448	0.822	2.552	0.200
APACHE II diagnosis group	1.000	0.998	1.001	0.546
Operative admission	1.342	0.797	2.259	0.268
Emergency admission	1.607	0.628	4.111	0.322
DIC	1.537	0.633	3.731	0.342
SAPS II score without age points	1.031	1.011	1.051	0.002
SOFA score, maximum during ICU stay	1.219	1.114	1.333	<0.001
Highest lactate	0.997	0.939	1.058	0.913
Female gender	0.785	0.495	1.244	0.303

Included patients 571 (132 in Q1 and 468 in Q2 to Q4). Hosmer-Lemeshow Chi-square 5.407, *P* = 0.713. RBC, red blood cell; Q, quartile; APACHE, acute physiology and chronic health evaluation; DIC, disseminated intravascular coagulopathy; SAPS, simplified acute physiology score; SOFA, sequential organ failure assessment.

## Discussion

In this large, observational multicenter study, we found that transfusion of older RBCs was independently associated with an increased risk of hospital mortality, but not 90-day mortality, or the development of KDIGO stage 3 AKI. The number of transfused RBC units, however, was independently associated with 90-day mortality.

Evidence on the impact of storage lesions of RBCs in critically ill patients is accumulating. The evidence for a deleterious effect arises mostly from observational studies. The only large RCT has been conducted in premature infants, and in this patient group, there was no morbidity or mortality benefit of fresher (less than 7 days) RBC transfusion compared to standard care [11]. The RCTs including adult patients have been smaller, with only 17 to 100 patients, aiming to test the feasibility of study logistics, or with surrogate variables as study endpoints [8-10,18]. Large RCTs are underway, but the results will be available only after several years [19,20], leaving clinicians uncertain with regard to the importance of age of RBCs.

RBC transfusions have also been associated with an increased risk of renal failure [21]. Transfusion of older RBCs has been associated with increased risk of renal failure in some studies [22,23], whereas in others no independent association of RBC age with AKI was found [21]. The power of our study was inadequate to study the association between age of blood and renal non-recovery. In the model predicting KDIGO stage 3, only scores on SAPS II without age points, and number of RBCs transfused, were independent predictors of AKI, whereas the age of RBCs was not. The prediction of KDIGO stage 3 AKI was also relatively inaccurate in this patient population as the median time from ICU admission to KDIGO stage 3 AKI was only 26 hours, and accordingly only a small proportion of RBC’s had been transfused prior to AKI.

Most observational studies on the effects of age of transfused RBCs on patient morbidity and mortality have been conducted in trauma and cardiac surgery patients [7]. Although these patients are often treated in the ICU, they have lower overall mortality as a group, and accordingly are not representative of the general critically ill patient population [24,25]. Of the two observational studies that have been conducted in unselected critically ill patients [17,26], one has been published only as an abstract [26]. In the Australian and New Zealand Intensive Care Society (ANZICS) study, the results were in line with our findings, as the hospital mortality increased with increasing age of the oldest RBC unit [17]. The long-term mortality was not, however, reported in that study. In a meta-analysis combining patients with various conditions (cardiac surgery, trauma and general hospitalized patients), transfusion of older RBC units was associated with increased mortality [7]. The highest impact on the result comes, however, from a retrospective study of 387,130 hospitalized patients, reporting one-week mortality. Critically ill patients represented less than 1% of the total number of patients in this meta-analysis [7].

There are several methods of producing RBC units from donated blood [13,27]. The differences in RBC production methods have an effect on the degree and magnitude of storage lesions over time, leading to variations in the recommended storage times for RBC units [27]. Interestingly, studies conducted in North America consistently report an increased risk of adverse endpoints with increased RBC storage age [28]. The results of studies conducted elsewhere show more variation [17,28]. Some of the differences in study results are suggested to be the result of a different production process leading to differences in storage lesions in RBC units [28].

This is the first study to describe the incidence of AKI and long-term mortality in critically ill patients transfused with RBCs of varying storage times. In our study, AKI was described by the latest recommended staging by KDIGO [15]. As presented in Table 2, there were seemingly more AKI and more stage 3 AKI in patients in the RBC age quartiles 2 to 4. However, when appropriate adjustments in multivariable analysis were performed, the age of transfused RBCs was not associated with the development of AKI stage 3. This implies that the incidence of KDIGO stage 3 AKI is not increased by transfusion of older RBCs. Hospital and 90-day crude mortality rates were significantly different between RBC age quartiles 1 versus quartiles 2 to 4. Here again, the adjustment for other variables showed that the transfusion of fresher RBCs was associated with hospital mortality but not 90-day mortality. The association between transfusion of aged RBCs and hospital mortality has been shown previously, but this is the first study to investigate the association with long-term mortality.

In non-randomized studies, there are frequently baseline differences between patient populations. In baseline variables, there are many differences in patient characteristics across quartiles of RBC age with increasing severity towards Q4. Previously, increased number of RBC transfusions has been associated with both sicker patients and increased risk of having older RBCs [29]. Adjusting for these differences in multivariable analysis translates findings more reliably, however, there is still room for residual confounders. This underlines the need for an RCT with sufficient power and clinically relevant endpoints to either confirm or refute the possible negative effects of older RBCs transfused to critically ill patients.

Our study has some limitations. First, the RBC data prior to ICU admission was not available, except for the information on massive blood transfusions (>10 units of RBCs in 24 hours) prior to admission. As individual RBC-unit information was not available, the age of RBCs in these pre-ICU transfusions remains unknown. Second, we did not collect the information on the use of other blood products, as the primary study was targeted to evaluate the incidence and prognosis of AKI in critically ill patients. Third, renal non-recovery was rare, and accordingly, the study was not powered to evaluate the possible associations of risk factors with poor renal recovery. Fourth, due to the observational design of this study, the transfused patients were different between quartiles of oldest RBCs transfused and those who were transfused versus non-transfused. In the analysis without adjustment for baseline differences, there was an increase in the incidence of AKI, and hospital and 90-day mortality (Tables 2 and 4). However, when adjusted for the baseline differences in multivariable analysis, the age of the oldest RBC unit was independently associated with hospital mortality only, but not with KDIGO stage 3 AKI or 90-day mortality. As we could adjust only for the variables that were collected in the study, residual confounders cannot be excluded.

## Conclusions

In this prospective observational study we were able to confirm the independent association between transfusion of older RBCs and increased risk of hospital mortality, but did not detect an independent association with 90-day mortality or the development of KDIGO stage 3 AKI. However, the number of transfused RBC units was an independent predictor of 90-day mortality in unselected critically ill patients. A large-scale multicenter RCT is needed to evaluate the possible negative effect of older RBCs in critically ill patients.

## Key messages

• 36.6% of critically ill patients were transfused during the ICU stay.

• Transfused patients received a median of 3 units (IQR 2 to 6) of RBCs.

• 70.2% of all transfusions were given within 72 hours of ICU admission.

• The age of transfused RBCs was independently associated with hospital mortality but not with 90-day mortality or KDIGO stage 3 AKI.

• The number of transfused RBCs was an independent risk factor for 90-day mortality.

## Abbreviations

AKI: Acute kidney injury; APACHE: Acute physiology and chronic health evaluation; DIC: Disseminated intravascular coagulopathy; FINNAKI: FINNish Acute Kidney Injury; KDIGO: Kidney disease: improving global outcomes; Q1: Lowest quartile; RBC: Red blood cell; RCT: Randomized controlled trial; RRT: Renal replacement therapy; SAPS II: Simplified acute physiology score; SOFA: Sequential organ failure assessment.

## Competing interests

The authors declare no competing interests on this study.

## Authors’ contributions

KMK: contribution to study conception and design, financing, acquisition of data, analysis and interpretation of data, drafting the article and responsible for the final output of the publication. SV: contribution to study conception and design, acquisition of data, statistical analysis and interpretation of data, drafting the article, intellectual contribution to study result analysis and critical revision of the manuscript. VP: substantial contributions to conception and design, financing, acquisition of data, analysis and interpretation of data, critical revision for important intellectual content of the manuscript. RB: Intellectual contribution to study result analysis and critical revision of the manuscript. JT: acquisition of data, analysis and interpretation of data, critical revision for important intellectual content of the manuscript. JC: analysis and interpretation of data, critical revision for important intellectual content of the manuscript. TK: analysis and interpretation of data, critical revision for important intellectual content of the manuscript. AK: acquisition of data, drafting the article, intellectual contribution to study result analysis and critical revision of the manuscript. MR: acquisition of data, intellectual contribution to study result analysis and critical revision of the manuscript. JK: contribution to study conception and design, acquisition of data, statistical analysis and interpretation of data, drafting the article, intellectual contribution to study result analysis and critical revision of the manuscript. AU: contribution to study conception and design, acquisition and interpretation of data, drafting the article, intellectual contribution to study result analysis and critical revision of the manuscript. The FINNAKI study group: contribution to study conception and design, financing, acquisition of data. All authors have approved the final version of the manuscript.

## Supplementary Material

Additional file 1: Table S1 Characteristics of non-transfused and transfused patients. **Table S2:** Times for ICU admission and AKI (acute kidney injury). **Table S3:** Odds ratios with 95% CI from logistic regression analysis of hospital mortality. **Table S4:** Cox regression analysis for 90-day mortality (enter method). Click here for file
