# Supplementary material for: Age of red blood cells and outcome in acute kidney injury
Source: Crit Care. 2013 Oct 4;17(5):R222. doi: 10.1186/cc13045 (PMC4057274; doi:10.1186/cc13045)
Supplement: Additional file 1: Table S1 — Characteristics of non-transfused and transfused patients. Table S2: Times for ICU admission and AKI (acute kidney injury). Table S3: Odds ratios with 95% CI from logistic regression analysis of hospital mortality. Table S4: Cox regression analysis for 90-day mortality (enter method). [file cc13045-S1.pdf]

Additional file Table S1: Patient characteristics of non-transfused and transfused patients

	**Non-transfused** (n=1146)	**Transfused** (n=652)	**P-value**
**Age, years**	61 [48-72]	66 [56-75]	<0.001
**Male gender**	741 (64.7%)	411 (63.0%)	0.506
**Operative admission**	309 (27.0%)	331 (50.8%)	<0.001
**Emergency admission**	1060/1142 (92.8%)	514/642 (80.1%)	<0.001
**Emergency surgery <1 week**	228 (19.9%)	187 (28.7%)	0.201
**Cardiac or vascular surgery**	102 (8.9%)	170 (26.1)	<0.001
**Trauma**	72 (6.3%)	44 (6.7%)	0.691
**Severe sepsis**	319 (27.8%)	222 (34.0%)	0.006
**DIC**	27/1116 (2.4%)	46/629 (7.3%)	<0.001
**Acute kidney injury**	366 (31.9%)	309 (47.4%)	<0.001
**Renal replacement therapy**	68 (5.9%)	96 (14.7%)	<0.001
**SAPS II score**	35 [26-48]	37 [29-50]	<0.001
**Maximum SOFA score***	7 [4-9]	8 [6-11]	<0.001
**Lactate** (mmol/L)**	2.1 [1.3-3.7]	2.40 [1.44-4.41]	<0.001
**Massive transfusion pre-ICU†**	22 (1.9%)	39 (6.0%)	<0.001
**Admission hemoglobin (g/L)‡**	120 [107-133]	98 [88-110]	<0.001

Data are expressed as median [IQR] or count (percentage)

DIC Disseminated Intravascular Coagulopathy, IQR Interquartile Range

*Data missing for 1 transfused patient

** Highest lactate 24 h preceding ICU admission or on first ICU treatment day. Data missing for 264 non-transfused and 51 transfused patients.

†Transfusion >10 RBC units in 24 hours

‡Data missing for 130 non-transfused and 77 transfused patients.

ICU intensive care unit, DIC disseminated intravascular coagulopathy, SAPS II Simplified Acute Physiology Score, SOFA Sequental Organ Failure Assessment

Additional file Table S2: Times for ICU (Intensive Care Unit) admission and AKI (Acute Kidney Injury).

	Q1	Q2	Q3	Q4	P
**no of patients with AKI**	52	78	90	89	
**Time from ICU admission to first AKI (hours)**	17.2 [8.0-32.0]	11.0 [5.8-25.7]	10.9 [5.0-29.4]	8.5 [1.4-20.3]	0.052
**Time from ICU admission to highest AKI (hours)**	19.4 [12.2-35.6]	20.5 [6.7-39.2]	22.6 [8.8-41.1]	14.8 [5.3-42.9]	0.531
**Time from ICU admission to first RBC (days) median (IQR)**	0 [0-1]	0 [0-1]	0[0-2]	0[0-2]	0.625
**Time from ICU admission to first RBC (days) mean (SD)**	0.94 (1.9)	1.1 (2.1)	1.4(2.5)	1.5 (3.5)	
**Time from hospital admission to ICU admission (days) median (IQR)**	1 [0-2]	1 [0-2]	1 [0-2]	1 [0-2.3]	0.957
**Time from hospital admission to ICU admission (days) mean (SD)**	5.0 (31.2)	5.5 (30.5)	4.4 (27.6)	3.5 (10.2)	

Additional file Table S3: Odds ratios with 95% Confidence Intervals of logistic regression analysis for hospital mortality

		95% Confidence Interval		
**Variable**	**Odds ratio**	**Lower**	**Upper**	**P-value**
Age	1.036	1.015	1.057	0.001
Propensity score for receiving >14 days old RBC	0.693	0.023	20.591	0.832
Number of transfused units	1.013	0.978	1.050	0.466
Acute kidney injury	0.93	0.513	1.686	0.811
Severe sepsis	0.881	0.491	1.580	0.671
RBC age Q 2-4 vs. Q1	2.157	1.023	4.546	0.043
APACHE II diagnosis group	1.001	0.999	1.003	0.354
Operative admission	1.539	0.817	2.898	0.182
Emergency admission	2.394	0.671	8.540	0.178
DIC	1.632	0.68	3.915	0.272
SAPS II score without age points	1.031	1.009	1.054	0.005
SOFA score, maximum during ICU stay	1.213	1.093	1.345	<0.001
Highest lactate	1.054	0.993	1.120	0.085
Female gender	0.864	0.504	1.482	0.596

Included patients 571 (132 in Q1 and 468 in Q2-4)

Hosmer-Lemeshow Chi-square 7.781, P=0.455

Additional file Table S4: Cox regression analysis for 90 –day mortality (enter method)

		95% confidence interval for HR		
**Variable**	**Hazard ratio, HR**	**Lower**	**Upper**	**P-value**
Age	1.030	1.017	1.043	<0.001
Propensity score for receiving >14 days old RBC	0.268	0.033	2.196	0.22
Number of transfused units	1.021	1.004	1.040	0.019
Acute kidney injury	0.828	0.563	1.219	0.339
Severe sepsis	0.863	0.608	1.226	0.411
RBC age Q2-4 vs.Q1	1.182	0.757	1.847	0.462
APACHE II diagnosis group	1.000	0.999	1.001	0.707
Operative admission	1.266	0.861	1.862	0.231
Emergency admission	1.480	0.657	3.334	0.345
DIC	1.073	0.649	1.775	0.783
SAPS II score without age points	1.024	1.010	1.037	0.001
SOFA score, maximum during ICU stay	1.163	1.092	1.239	<0.001
Highest lactate	1.008	0.971	1.046	0.68
Female gender	1.161	0.828	1.627	0.386
